# Serological investigation of some important RNA viruses affecting sheep and goats in Giza and Beni-Suef governorates in Egypt

**DOI:** 10.14202/vetworld.2017.1161-1166

**Published:** 2017-10-02

**Authors:** Mohamed Abd El-Fatah Mahmoud, Mohamed Karam Elbayoumy, Doaa Sedky, Sahar Ahmed

**Affiliations:** 1 Department of Parasitology and Animal Diseases, Division of Veterinary Research, National Research Centre, 12622 Dokki, Giza, Egypt; 2 Department of Cell Biology, Division of Genetic Engineering & Biotechnology, National Research Centre, 12622 Dokki, Giza, Egypt

**Keywords:** bluetongue, enzyme-linked immunosorbent assay, foot and mouth disease, goats, Peste des Petits ruminants, sheep

## Abstract

**Aim:**

The aim of this study was to investigate the seroprevalence of antibodies against foot and mouth disease (FMD), Peste des Petits ruminants (PPR), and bluetongue (BT) in sheep and goats within Giza and Beni-Suef governorates at the second half of 2016.

**Materials and Methods:**

A total of 300 animals (sheep and goats) randomly selected from small stocks with no history of previous vaccination against FMD virus (FMDV), PPR, or BT viruses (BTV) and examined with competitive enzyme-linked immunosorbent assay for detection of FMD-non-structural protein, PPR, and BT antibodies.

**Results:**

Seroprevalence analysis revealed that antibodies against FMDV were 40.8% and 37.1% at Giza governorate, while at Beni-Suef governorate, the percent was 36.7% and 50% in sheep and goat, respectively. Antibodies against PPR were 63.8% in sheep and 45.7% in goats at Giza governorate, whereas the results for Beni-Suef governorate were 71.7% in sheep and 45% in goats. Antibodies against BT were 45% and 37% in sheep and goats, respectively, in Giza governorate, whereas the results for Beni-Suef governorate were 80% and 55% in sheep and goats, respectively. The average of BTV antibody prevalence was significantly higher in sheep (45% and 80%) than in goats (37% and 55%) in Giza and Beni-Suef, respectively. Statistical analysis for the three viruses showed the high relation between the two governorates in case of sheep (r=0.85) and in case of goats (r=0.87). In general, a strong positive correlation was observed between the governorates (r=0.93).

**Conclusion:**

Giza and Beni-Suef governorates are endemic with FMDV, PPR, and BTV. Regional plan for characterization and combating FMD, PPR, and BT is recommended to help in the achievement of the most suitable combination of the vaccine regimen.

## Introduction

Small ruminants mainly sheep and goats contribute significantly to the economy of farmers in African and Asian countries. Sheep and goats are a source of meat, milk, and wool in addition to their rapid growth and reproduction. Poor man considers goats as cows in developing countries [1].

In Egypt, sheep and goats play a dynamic role in the economy of poor, destitute, and landless workers. Many viral diseases attack sheep and goats, namely, foot-and-mouth disease (FMD), bluetongue (BT) disease, maedi-visna, orf, tick-borne encephalomyelitis, Peste des Petits ruminants (PPR), sheep pox, and goat pox [2,3].

FMD virus (FMDV) is a positive, single-stranded RNA virus, a member of family Picornaviridae [4,5]. It is a highly transmissible disease of both wildlife and house-trained even-toed animals. More than 65 wild animal species are susceptible to FMD infection [6].

Serologically, seven serotypes of the virus were identified as O, A, Asia 1, C, SAT 1, SAT 2, and SAT 3, and each serotype has multiple subtypes [7]. The viral particles present in all discharges and secretions of sick animals, so the virus spreads efficiently. Infection occurs through the exposure to the contaminated materials either directly or indirectly [8]. Control of FMD infection is so difficult as the wind can spread the virus for a distance of 10 km [9]. There is no cross-protection against different FMD serotypes [10].

The disease characterized by a low mortality rate (5%) and high morbidity rate (100%) in adult animals. FMD is responsible for the production losses represented by low milk yield and weight loss [11].

FMDV genome is 8.5 kb naked RNA virus, and this genome codes for structural protein (SPs) and non-SPs (NSPs). Although, antibodies against both SPs and NSPs could be detected in infected animals, and antibodies against NSPs are not present in vaccinated not infected animals [12]. Hence, in using kits that can identify antibodies against NSPs, we can differentiate diseased animals from vaccinated one [13].

PPR causes discrete financial troubles in sheep and goats farms [14]. PPR virus attacks sheep and goats and leads to pneumoenteritis [15]. PPR virus is a member of genus morbillivirus, Paramyxoviridae [16]. Four lineages (I, II, III, and IV) of PPR virus were identified on sequencing of the fusion (F) protein [17]. Epidemiologically, PPR prevails mainly in Africa and Asia [18]. The PPR viral particles present in all secretions and discharges of the diseased animal [19]. The disease causes high morbidity and mortality rates reach to 100% in highly susceptible animals [20].

BT is an infectious arthropod-borne viral disease of sheep and goats. BT virus (BTV) has an RNA genome of double-stranded nature (genus Orbivirus and family Reoviridae) which attacks housetrained and wild ruminants [21]. 24 separate BTV serotypes have been recognized for decades, and all of them can initiate the infection in ruminants. However, two new BTV serotypes, BTV-25 (Toggenburg orbivirus, from Switzerland) and BTV-26 (from Kuwait), were recently registered in goats and sheep, respectively [22].

The disease affects fine wool and mutton breeds of sheep severely; cattle represent the chief mammalian reservoir of the BTV and play a very important role in the epidemiology of the disease [23]. The virus infects goats and wild ruminants but generally with mild or no clinical signs. Culicoides mainly transmit BTV beside the oral and vertical route in sheep and cattle. Epidemiologically, BT disease is common in humid areas of the world with no clear symptoms in the native sheep populations [24]. The distribution of BT is constrained to a latitudinal band around the world between 50°N and 30°S of the world where Culicoides midges are tremendously abundant [25].

Clinical signs of the sickness are usually clear in sheep and some uninhabited ruminants but are rare in goats and cattle. Symptoms vary from subclinical to acute febrile response; it causes facial edema and hemorrhages, ulceration of the mucous membranes. There is often severe muscle degeneration and skeletal myopathy [26].

In Egypt, BTV infection was recognized for the first time in foreign Marino sheep [27]. The identified BTV serotypes in the succeeding epidemics were BTV 1, 4, 10, 12, and 16 [28]. Mahmoud and Khafagi [23] conducted a serosurvey on samples collected from 14 governorates of the upper and lower Egypt. About 17.5% of sheep and 14.7% of goats’ serum samples were positive. In all tested governorates, the prevalence of BT antibodies was 17.5% in sheep and 14.7% in goats. The overall prevalence of anti-BT antibodies in different governorates was 16.9%. Giza and Beni-Suef governorates recorded the highest prevalence of BT group specific antibodies with 30% (24/80) and 71.8% (74/103) in Giza and Beni-Suef governorates, respectively.

This study aimed to investigate the seroprevalence of antibodies against FMD, PPR, and BTVs in sheep and goats within Giza and Beni-Suef governorates. Examined sheep and goats express the environment of sheep and goat holders with small numbers, group-housed with cattle and buffaloes and not comply with any immunization programs even if present.

## Materials and Methods

### Ethical approval

Ethical approval from Institutional Animal Ethics Committee and local laws and regulations were considered in applying our experiment.

### Animals

A total of 300 serum samples from sheep and goats were collected from AL-Hawamdia district-Giza Governorate and Kafr Abo Qassim-Beni-Suef Governorate in the second half of 2016. Selected animals in this study reared in association with both buffaloes and cattle.

Animals were randomly selected from small stocks that do not apply vaccination against FMDV, PPR, and BTVs, and there is no history of previous vaccination. Types and numbers of animals examined in each governorate distributed according to Table-1.

**Table-1:** Seroprevalence against FMDV, PPR, and BTV in Giza and Beni-Suef governorates.

Governorates	Animal species	Total serum no.	Single infection			Double infection			Triple infection	Total positive samples to		
			
			FMD (%)	PPR (%)	BT (%)	FMD/PPR (%)	FMD/BT (%)	PPR/BT (%)	FMD/PPR/BT	FMD (%)	PPR (%)	BT (%)
Giza	Sheep	130	19 (14.61)	39 (30.0)	17 (13.07)	16 (12.3)	13 (10)	23 (17.69)	5 (3.8)	53 (40.77)	83 (63.84)	58 (44.61)
	Goats	70	9 (12.86)	12 (17.14)	7 (10.0)	7 (10)	6 (8.57)	9 (12.85)	4 (5.7)	26 (37.14)	32 (45.71)	26 (37.14)
	Total	200	28 (14.0)	51 (25.5)	24 (12.0)	23 (11.5)	19 (9.5)	32 (16)	9 (4.5)	79 (39.5)	115 (57.5)	84 (42.0)
Beni-Suef	Sheep	60	8 (13.33)	18 (30.0)	25 (41.67)	5 (8.3)	3 (5)	14 (23.33)	6 (10)	22 (36.66)	43 (71.66)	48 (80.0)
	Goats	40	11 (27.5)	8 (20.0)	13 (32.5)	4 (10)	3 (7.5)	4 (10)	2 (5)	20 (50.0)	18 (45.0)	22 (55.0)
	Total	100	19 (19.0)	26 (26.0)	38 (38.0)	9 (9)	6 (6)	18 (18)	8 (8)	42 (42.0)	61 (61.0)	70 (70.0)

FMDV=Foot and mouth disease virus, PPR=Pest des Petites ruminants, BTV=Bluetongue virus

### Serum preparation

Blood samples were collected aseptically from the jugular vein from each animal using plain vacutainer tubes. Serum was separated by centrifugation of the blood at 3000 rpm for 10 min at room temperature; aliquots were transferred into a 1.5 ml sterile microcentrifuge tube. All serum samples were stored at −20°C until used for a serological investigation [29].

### Detection of FMD NSP antibodies

Serum samples were used to monitor antibody against non-structural polyprotein (NSP) 3ABC of FMD antigen using marketable enzyme-linked immunosorbent assay (ELISA) kit (IDEXX FMD 3ABC Bo-Ov), and we followed the manufacturer instructions. According to the ELISA test kit manual, samples with percentage values >30% were considered positive, <20% as negative, and samples between 20% and 30% were considered suspicious [30].

### Detection of PPR antibodies using competitive ELISA (cELISA)

cELISA kit and its protocol provided by IAH (Pirbright Laboratory, UK). The test is depending on the competition between the monoclonal antibody (MAb) against the tested serum antibodies for binding to the H protein antigen [31]. Serum antibodies compete with the enclosed MAb to fix to the coated plate. The test was carried out according to the supplied protocol. Both the negative and positive cutoff values were utilized from the controls of the test procedure. Using Immunoskan reader produced by Flow Laboratories, UK, we read the ELISA plates at 492 nm wavelength filter. Calculation of the result was gotten automatically by the aid of installed software on a computer connected to the reader. This software is produced by FAO/IAEA, Vienna, Austria, and calculates percentage inhibition (PI) values directly. The optical density (OD) values could be changed to PI by the following formula:


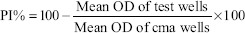


Where, OD=The optical density value, cma=The MAb control. Inhibition values more than 50% were considered positive.

### Detection of BT antibodies using cELISA

cELISA Kit for detection of BT antibodies produced by BDSL, Biological Diagnostic Supplies Ltd., Surrey, UK was used. It is designed to detect the antibodies against the VP7 antigen. The test was carried out according to the supplied protocol. The PI values were calculated according to the formula of the study by Afshar *et al*. [32]. Samples that give PIs ≥50% were considered positive, and those with PIs of <50% were negative.

### Statistical analysis

Pearson correlation coefficients were calculated according to http://www.socscistatistics.com/tests/pearson/Default2.aspx [33].

## Results and Discussion

Small ruminants’ livestock in Egypt is an easy and vulnerable target for many bacterial and viral epidemic diseases. Low efficiency existing control programs facilitate the spreading of different infectious and contagious diseases. A large number of viral diseases have the potential to cause serious losses in sheep and goats such as PPR, BT, and FMD.

In this study, we highlighted the serological prevalence of FMD, PPR, and BT in sheep and goats at Giza and Beni-Suef governorates, to clarify the epidemiological situation of the three viruses through evaluating its seroprevalence. Results of this study will be helpful for the decision-makers and stockholders.

**Figure-1:**
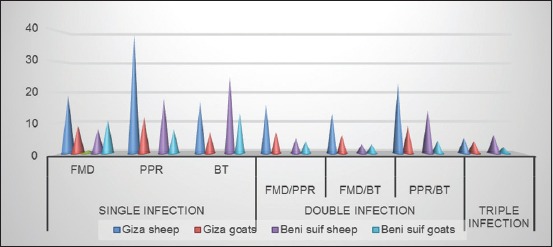
Comparison of single, double, and triple different viral infection in sheep and goats at Giza and Beni-Suef governorates.

As presented in Table-1 and illustrated in Figure-1, results revealed that the percent of serologically positive animals against FMDV was 40.8% and 37.1% at Giza governorate, whereas at Beni-Suef governorate, the percent were 36.7% and 50% in sheep and goat, respectively.

NSPs-FMD-dependent kits can identify antibodies to the NSP. Although this test can segregate the diseased animal from the immunized one, it cannot distinguish between the FMD serotypes [34].

Actually, buffaloes maintain FMD infections and infect other susceptible species in Sub-Saharan Africa [35]. Natural and experimental transmission of FMD from carrier buffalo to cattle was confirmed [8].

Lots of positivity to FMDV indicates that the disease is endemic in the two governorates and also indicate that there are no major alterations between the prevalence of illness in sheep and goats. In fact, farmers raise sheep and goats together, so they have the same chances to pick up FMD and this is may be the reason for the non-significant difference in the prevalence of antibodies to FMDV. Comparable disease form was also documented in different reports. In Southern Jordan, the seroprevalence rates of FMD in sheep and goat at individual species level was reported to be 10.4% and 6.3%, whereas seroprevalence at herd level was 44.7% and 33.3%, correspondingly [36]. In Uganda, a positivity of 14% and 22% in goats and sheep, respectively, was reported [37]. In Pakistan, another serological study to NS proteins of FMD reported a positivity of 19.44% and 21.27% in sheep and goats, respectively. The overall seroprevalence rate was 21%. The seroprevalence to the FMD-NSP in three different areas in Pakistan was 25.75%, 4.75%, and 32.5%. The environment in which sheep and goats were raised was poor and represents a stress factor on the animals. Bad housing and the nutritional deficiency facilitate the spread of contagious diseases [38].

The difficulty of FMD recognition in sheep and goats and its numerous mobilizations play a role in the spreading of the disease between the susceptible animals [39]. Low infection within these farms combined with the findings of previous reports poses that the infected sheep and goats represent only a limited threat for FMD spread [40]. In endemic countries, in which mass vaccination is applying to all livestock, sheep and goats have only a partial role in the spreading of FMD infection. Meanwhile, it is desirable to include sheep and goats in the vaccination programs in FMD endemic republics or in countries that proceed to eradicate FMD [41]. Complete identification of the circulating FMD viral strains is essential to achieve the appropriate vaccine formula of good effectiveness [24].

Sheep are more resistant to PPR virus than goats, so it mounts a more humoral immunity [42,43]. Affected sheep with PPR are usually apparently normal [44]. Goats have a high liability to PPR that leads to death; therefore, the number of serologically positive goats to PPR are usually small [45].

Results showed that the rates of antibodies in sheep and goats sera against PPR were 63.8% in sheep and 45.7% in goats at Giza governorate, whereas the results for Beni-Suef governorate were 71.7% in sheep and 45% in goats. This may be due to the closeness of these regions to many neighboring villages, where PPR is endemic, and to the unrestricted movement of relatively large numbers of sheep and goats from these “endemic” regions into the surrounding districts.

There were poor data for the serological status of BTV in Egypt. The first serological survey was conducted by Hafez and Ozawa [27]. The overall ratio of BTV antibodies was (9%) using the agar gel immune-precipitation test (AGPT), and the antibodies prevalence in ovine sera was 37%. Mahmoud and Khafagi [23] conducted a serosurvey on samples collected from 14 governorates of the upper and lower Egypt; the overall prevalence of BT antibodies was 17.5% in sheep and 14.7% in goats. The overall prevalence of anti-BT antibodies in different governorates was 16.9%. Giza and Beni-Suef governorates recorded the highest prevalence of BT group-specific antibodies, and in both governorates, the prevalence of BT antibodies was 30% (24/80) and 71.8% (74/103), respectively. In our study, antibodies prevalence in sheep and goats’ sera against BT was 45% and 37% in sheep and goats, respectively, in Giza governorate, whereas the results for Beni-Suef governorate were 80% and 55% in sheep and goats, respectively. There is a significant variation between our results and that obtained by the findings of Mahmoud and Khafagi [23], Hafez and Ozawa [27]. This variation could be due to the variation in the used techniques, in our study, we depend on the ELISA technique, which is more sensitive than the AGPT that used by Mahmoud and Khafagi [23], Hafez and Ozawa [27].

The average of BTV antibody prevalence was significantly higher in sheep (45% and 80%) than in goats (37% and 55%) in Giza and Beni-Suef, respectively; this is may be due to the low susceptibility of goats to natural infection with BT [46]. In addition, we selected sheep and goats mixed with cows and buffaloes. High prevalence of antibodies in the examined animals may be due to their presence next to cows, which is the main reservoir of the BTV. Mixed breeding between different species of livestock is an obstacle in the control operations and a means of transmitting diseases between these species [47].

The animal susceptibility to the virus infection is exaggerated by many epidemiological factors such as fitness, spreading of the vector and feeding behaviors of the vector, and older animals tend to be more liable than younger ones. The severity of symptoms looks to vary according to the animal breed and the viral serotype [48].

Statistical analysis showed a high correlation between the two governorates in case of sheep (r=0.85) and in case of goats (r=0.87). In general, a strong positive correlation was observed between the two governorates; the value of r=0.93. The value of r^2^, the coefficient of determination, is 0.8636.

The reported seroprevalence of FMD with BT in sheep and goats was high. Serotyping of the FMDV and BTVs within the Egyptian governorates specially Giza and Beni-Suef governorates is recommended. We concluded banning of importation from the endemic areas by such viruses, in addition to respectable checking and accreditation at the quarantine checkpoints.

As shown in Table-1 and illustrated in Figure-1, results showed that PPR/BT represents the highest percentages, while the FMD/BT represents the lowest one when compared with the corresponding total numbers. FMD/BT showed moderate percentages. These results indicate that PPR and BT are endemic in the two governorates more than FMD. In each governorate, the antibodies prevalence PPR/BT is more in sheep than in goats. This is due to the high susceptibility of goats to PPR than sheep. In comparison with the two governorates, Giza possesses a higher percentage of antibodies prevalence against PPR/BT (12.85%) than Beni-Suef (10%) in goats. Giza possesses a lower percentage of antibodies prevalence against PPR/BT (17.69%) than Beni-Suef (23.33%) in sheep. This result could be interpreted, as at the individual level, the total prevalence of antibodies against both PPR and BT was higher in Beni-Suef (61% and 70%) than in Giza (57.5% and 42%) for sheep and goats collectively.

Results showed large number of positive sheep (10%) to FMD/PPR/BT in Beni-Suef than Giza (3.8%). Giza possesses a higher percentage of antibodies against FMD/PPR/BT (5.7%) than Beni-Suef (5%) in goats. The total positive sheep and goats to FMD/PPR/BT were lower in Giza (4.5%) than in Beni-Suef (8%).

The maximum single seroprevalence was in PPR infection 39/130 (30%) and the maximum double seroprevalence was observed in PPR/BT infection 23/130 (17.69%) as shown in Table-1.

## Conclusion

The examined governorates were endemic with FMD, PPR, and BT. Therefore, sheep and goats may transmit such viruses to contact farm animals and remain infective for long time without detectable clinical signs. Thus, sheep and goats may play a role in the persistence and transmission of different viral infection.

Regional plan for combating FMD is recommended. Full characterization of FMD strains is important, as it helps in the achievement to the most suitable combination of the vaccine formula. Sheep and goats must be included in FMD vaccination program. Mixed breeding between different species of livestock is an obstacle in the control operations and a means of transmitting diseases between these species such as BT. Sheep and goats may pick up the infection due to their presence next to cows, which are the main reservoir of the BTV. We are in need for further new studies for identification and characterization of the circulating BT strains in Egypt. Moreover, special attention should be considered to avoid importation of animals from the countries in which recent outbreaks of FMD, PPR, and BT occurred.

## Authors’ Contributions

MAEM, MKE, DS, and SA conceived the study, carried out the laboratory work, and analyzed the data. MAEM performed the fieldwork and collected the samples. MAEM and MKE drafted the manuscript. All authors read and approved the final manuscript.
